# A Life Events Scale for Armed Forces personnel

**DOI:** 10.4103/0019-5545.31580

**Published:** 2006

**Authors:** Suprakash Chaudhury, Kalpana Srivastava, M.S.V. Kama Raju, S.K. Salujha

**Affiliations:** * Associate Professor, Presently Professor and Head, Department of Psychiatry, RINPAS; ** Scientist ‘E’ (Clinical Psychologist), Department of Psychiatry, Armed Forces Medical College, Pune 411040, Maharashtra; *** Professor and Head, Department of Psychiatry, Armed Forces Medical College, Pune 411040, Maharashtra; **** Associate Professor, Department of Psychiatry, Armed Forces Medical College, Pune 411040, Maharashtra

**Keywords:** Stressful life events, assessment, AFMC LES

## Abstract

**Background:**

Armed Forces personnel are routinely exposed to a number of unique stressful life events. None of the available scales are relevant to service personnel.

**Aim:**

To construct a scale to measure life events in service personnel.

**Methods:**

In the first stage of the study open-ended questions along with items generated by the expert group by consensus method were administered to 50 soldiers. During the second stage a scale comprising 59 items and open-ended questions was administered to 165 service personnel. The final scale of 52 items was administered to 200 service personnel in group setting. Weightage was assigned on a 0 to 100 range. For normative study the Armed Forces Medical College Life Events Scale (AFMC LES) was administered to 1200 Army, 100 Air Force and 100 Navy personnel.

**Results:**

Service personnel experience an average of 4 life events in past one year and 13 events in a life-time. On an average service personnel experience 115 life change unit scores in past one year and 577 life change unit scores in life-time on the AFMC LES. The scale has concurrent validity when compared with the Presumptive Stressful Life Events Scale (PSLES). There is internal consistency in the scale with the routine items being rated very low. There is a pattern of uniformity with the civilian counterparts along with differences in the items specific to service personnel.

**Conclusions:**

The AFMC LES includes the unique stresses of service personnel that are not included in any life events scale available in India or in the west and should be used to assess stressful life events in service personnel.

## INTRODUCTION

Stress is a normal part of human existence—a double-edged sword, which can help us mobilize and achieve, or physically and psychologically incapacitate us. War is the most dramatic concentration of deliberate physical and psychological trauma that societies can inflict upon each other. Under some combat conditions not only elevated symptom levels but also breakdown in performance became endemic.^1^ In a study of 2630 soldiers who had broken down during combat in Normandy campaign in World War II, it was estimated that the onset of combat exhaustion occurred even in previously normal soldiers when about 65% of their companions had been killed, wounded or had otherwise become casualties.^2^ The extent to which symptoms produced in extreme situations in previously normal persons are transient and self-limiting is a matter of controversy. Against the background of exposure to the brutalities of Nazi concentration camps, there is strong evidence that not only has severe stress-induced psycho-pathology persisted in survivors but also that the survivors are more prone to physical illness and early death.^1^ Similarly, 17% of the US population outside of New York City reported symptoms of September 11-related post-traumatic stress 2 months after the attacks; 5.8% did so at 6 months.^3^ Natural and man-made disasters, fortunately, are rare occurrences whose devastating effects are limited to relatively small populations of exposed persons. Yet psychopathology and somatic disturbances are far from rare in peace-time populations relatively secure from war, flood, and other disasters. If stressful situations play an aetiological role in these disorders the events involved must be more ordinary, more frequent experiences in the lives of most people—things such as marriage, birth of a first child, and death of a loved one. Based on this hypothesis stressful life events scales have been constructed. A large number of published reports attest to the association of life stress and a wide range of physical and psychiatric disorders.^4^–^12^

Due to cultural variations the popular stressful life events scales constructed in the West^13^,^14^ are not valid for other countries and the trend has been to construct life events specifically for different populations including India.^15^,^16^ However, these scales have limited utility for measuring life events in armed forces personnel because a number of stressful life events such as fighting against enemies, fighting against terrorists, posting to field or operational areas are unique to service personnel. Obviously, these stressful life events are not present in scales that have been constructed keeping the civilian population in mind. Moreover, certain events considered as stressful, viz. exposure to disasters and mass casualties, frequent change of residence of self and family are commonly experienced by armed forces personnel. It is likely that their appraisal of these events may differ from that of the civilian population. Thus, it is evident that in respect of Indian Armed Forces personnel there is no scale available to measure stressful life events. In view of the above, the present study was undertaken to make available for the first time a suitable scale to measure stressful life events in Indian armed forces personnel.

## METHODS

### Construction of the Armed Forces Medical College Life Events Scale

A pilot study was conducted on 50 soldiers. Initially items for the scale (*n* = 87) were taken from consensus of experts in the field. Face validity of items was taken into consideration and items were drawn from the common experience of service personnel. After initial analysis some items were excluded from the list. For example, some of the items of routine activities such as physical training, order to come on duty, which are daily occurrences in the Armed Forces, were excluded. Some items were grouped. For example death of father, death of mother, death of grandparents was combined to death of near and dear ones. The second version of the scale contained 59+3 items. One item was repeated for internal consistency of the item. At the end 3 open-ended questions were also included to enable respondents to report any other stressful event, which was not included in the scale.

This scale was administered to 165 service personnel drawn from the local garrison. Men belonging to various arms and services, and different trades were randomly selected for the study. The criteria for inclusion were minimum educational level of matriculation and no history of physical or mental illness in the past one-year. Standard instructions were given to the soldiers in Hindi and English. They were told that results would be confidential. To maintain secrecy of the identity, code numbers were assigned and the same numbers were written on the forms. Names of the personnel were not endorsed. Standard instructions to assign weightage to each item were given with suitable examples in the range of 0 to 100. Routine items involving no significant change had a weightage of 0, while an event, which is considered to be having the highest change had a weightage of 100. Each individual was asked to give his own assessment of each event irrespective of having experienced the event or not. Next they were asked to mark the event whether it has occurred within a year or earlier in his life-time. Data so generated were analysed by an appropriate statistical method.^17^ Based on the results 10 items (6 items assigned lowest life change score, 1 duplicated item and 3 open-ended questions) were dropped and 2 items were modified. The final version of the scale contained 52 items and was named the Armed Forces Medical College Life Events Scale (AFMC LES) (*see* Appendix A, page 176).

The AFMC LES was administered in group settings to a randomly selected sample of 200 servicemen belonging to various arms and services, and different trades, from all personnel posted to Pune, Kirkee and Lonavala. The criteria for inclusion were minimum educational level of matriculation, no history of physical illness in the past one year and no life-time history of mental disorders. Confidentiality was ensured by not recording personal identification data such as name, trade and unit. Standard instructions were given for the assessment of life change units. Each individual was asked to give his own assessment of each event irrespective of having experienced the event or not. Next they were asked to mark the event whether it had occurred within a year or earlier in their life-time. They were also asked to mark whether they considered each event as desirable or undesirable. The individuals were approached again after 1 week for reassessment of weightage given to the items to establish reliability. The mean scores assigned to each event by the respondents were the life change unit (LCU) score for that event. Data so generated were statistically analysed to arrive at a weighted mean life change score in respect of each item. The Presumptive Stressful Life Events Scale (PSLES) was administered to all the subjects in the same sitting when the AFMC LES was filled for determining the concurrent validity of the latter.

*Norms for the number of experienced life events:* The AFMC LES was administered under supervision of a Graded/ Classified Specialist in Psychiatry to 1200 Army personnel posted in peace and field areas including counter-insurgency operational areas (Pune, Srinagar, Udhampur, Chandigarh, Lucknow, Jodhpur, Binaguri, Tezpur, Guwahati and Jorhat), 100 Air Force personnel (Pune, Bangalore, Guwahati, Tezpur, Srinagar, and Jorhat) and 100 Navy personnel (Pune, Mumbai, Vishakapatnam and Cochin). All subjects were free from medical or psychiatric illnesses. Random sampling procedure was used to extract the sample. Personal identification data such as name, trade and unit of the personnel were not endorsed to ensure confidentiality. The forms were scored centrally at AFMC. Results were tabulated and statistically analysed using SPSS utilizing parametric tests for continuous data and non-parametric tests for ordinal data. Cornbach's alpha, split-half reliability, test–retest reliability, were calculated using the SPSS software package.^18^

*Factor analysis:* With the set of scores on the 52 life change events, the correlation between them was calculated to yield a correlation matrix. Factor analysis using the SPSS statistical software was used to simplify the correlation matrix and identify the smaller number of factors which could explain the correlation. A component or a factor explains the variance in the intercorrelation matrix, and the amount of variance explained is known as the eigenvalue for the factor. A factor loading is the correlation of a variable with a factor. A loading of 0.3 or more is frequently taken as meaningful when interpreting a factor. In the present study we used a loading of 0.3 or more as significant cut-off value except for one item which had a loading of 0.290. An exploratory factor analysis was carried out by first performing a principal component analysis. The number of factors was determined by using the cut-off eigenvalue = or > 1 and also cross-validated by the scree plot. The analysis was run with rotation of factors using the Varimax method.^18^

## RESULTS

### Quantification of stressful life events

The mean LCU scores for the 52 events ranged from 83 for spouse having illicit relations to 23 for going on posting (Table 1). The life events deemed to be desirable or undesirable by the majority of service personnel in the study are shown in Table 2.

**Table 1:** Stressful life events in service personnel and life change unit (LCU) score

Item No.	Life event	LCU score		
		
		MeanSD SEM		
1	Spouse having illicit relations	83	28.34	2.004
2*	Court martial	81	26.10	1.846
3	Amputation of body parts	80	24.20	1.711
4	Divorce	78	22.76	1.610
5	Going abroad on duty	72	21.33	1.508
6*	Receiving medal for bravery	71	28.68	2.028
7*	Fighting against enemies during war	69	23.42	1.656
8*	Loss of identity card	69	30.69	2.170
9	Child getting a job	68	26.47	1.872
10	Getting married	67	28.03	1.982
11	Hospitalization due to serious illness	65	21.36	1.511
12	Winning a lottery	64	28.12	1.989
13	Constructing own house	63	23.15	1.637
14	Birth of child	62	28.26	1.999
15*	Going on posting within 48 hours	62	30.14	2.132
16*	Fighting against terrorist	61	26.71	1.889
17	Conflict with family members	59	27.32	1.932
18	Sex related problems	58	32.19	2.277
20	Sanctioned leave being cancelled	56	31.14	2.202
21	Demotion	56	29.32	2.074
22*	Red ink entry	54	31.80	2.249
23	Wife not conceiving for long duration	54	28.13	1.989
24	Getting release from service	53	32.73	2.315
25	Child leaving town for higher education	53	28.27	1.999
26	Child not getting admission in school	53	27.36	1.935
27	Spending tenure of high altitude posting	51	26.36	1.864
28	Completing a tenure in operational area	50	27.81	1.967
29	Arranging for a big loan	49	26.52	1.876
30	Marriage of daughter	49	27.32	1.932
31	Change of trade	49	25.61	1.811
32	Receiving medals in sports	47	28.69	2.029
33	Dowry related problems in family	47	26.19	1.852
34	Not receiving salary because of debit	46	27.43	1.940
35*	Completing a tenure of field posting	46	29.34	2.075
36	Shifting house many times in same station	46	27.61	1.953
37	Sanction of casual leave	44	23.06	1.631
38	Not getting government accommodation	43	31.44	2.183
39	Wife starting a job	42	23.16	1.638
40*	Pay fine	40	28.13	1.989
41	Difficulty with seniors	39	31.38	2.219
42	Annual leave not being sanctioned	39	27.16	1.921
43	Conflict with friends in unit	38	27.37	1.936
44	Passing the promotion cadre	38	26.23	1.855
45	Receiving highest marks in firing	38	26.11	1.847
46	Black ink entry	37	21.16	1.496
47	Wife leaving the job	37	27.46	1.942
48	Lack of son	37	29.91	2.115
49	Participation in divisional exercises	36	25.34	1.792
50	Failing in promotion cadre	36	30.21	2.137
51	Sanction of annual leave	34	26.45	1.871
52	Going on posting	23	21.72	1.536

* Life stress items unique to service personnel

**Table 2:** List of desirable and undesirable items of the AFMC Life Events Scale

	Desirable life event		Undesirable life event
1.	Getting married	1.	Shifting house many times in same station
2	Birth of child	2.	Loss of identity card
3.	Child getting a job	3.	Child not getting admission in school
4	Passing the promotion cadre	4.	Wife not conceiving for long duration
5	Completing a tenure in operational area	5.	Lack of son
6	Participation in divisional exercises	6.	Wife/husband having illicit relations
7	Completing a tenure of field posting	7.	Wife leaving the job
8	Child leaving town for higher education	8.	Sex-related problems
9	Wife starting a job	9.	Failing in promotion cadre
10.	Receiving medal in sports	10.	Sanctioned leave being cancelled
11.	Receiving medal for bravery	11.	Annual leave not being sanctioned
12.	Going on posting	12.	Divorce from wife
13.	Winning a lottery	13.	Conflict with family members
14.	Sanction of casual leave	14.	Not receiving salary because of debit
15.	Arranging for a big loan	15.	Court martial
16.	Spending tenure of high altitude posting	16.	Black ink entry
17.	Change of trade	17.	Pay fine
18.	Marriage of daughter	18.	Hospitalization due to serious illness
19.	Constructing own house	19.	Death of a close relative
20.	Fighting against enemies during war	20.	Red ink entry
21.	Fighting against terrorists	21.	Not getting government accommodation
22.	Going abroad on duty	22.	Going on posting within 48 hours
23.	Getting release from service	23.	Demotion
24.	Receiving highest marks in firing	24.	Difficulty with seniors
25.	Sanction of annual leave	25.	Conflict with friends in unit
		26.	Dowry-related problems in family
		27.	Amputation of body parts

### Cross-cultural comparison of the life events

A comparison of the AFMC LES with Holmes and Rahe's Social Readjustment Rating Scale (SRRS) and the PSLES revealed that life event items of AFMC LES overlap with SRRS and PSLES only to a modest degree. Twenty-nine of the 52 AFMC LES items (55.8%) were not included in the SRRS. Similarly 24 of the 52 AFMC LES items (46.2%) were not included in the PSLES. The mean LCU scores of life events, which are common to the AFMC LES along with the scores on similar items on the PSLES, SRRS, Paykel's New Haven Life Events Measure and Zhou and Lin's scale are shown in Table 3.

**Table 3:** Mean life change unit (LCU) scores of common stressful life events on AFMC LES compared with scores on similar items on other life event scales (Indian and Western)

Life event	LCU scores				
	
	AMC LES	PSLES	SRRS	Paykel*	Zhou & Lin**
Spouse having illicit relations	83	80	-	16.78	-
Suspension/court martial	81	76	-	17.61	-
Divorce	78	77	73	16.00	3.08
Getting married	67	43	50	5.61	-
Hospitalization due to serious illness	65	56	53	14.61	3.42
Constructing own house	63	46	-	-	-
Birth of child	62	30	-	-	-
Conflict with family members	59	47	-	12.11	2.19
Sex-related problems	58	51	39	-	2.39
Death of a close relative	58	66	63	17.21	3.51
Demotion	56	-	-	15.05	-
Wife not conceiving for long duration	54	67	-	-	-
Getting release from service/retirement	53	35	45	9.33	-
Child leaving town for higher education	53	55	29	7.20	-
Child not getting admission in school	53	-	-	-	2.55
Arranging for a big loan	49	49	30	12.64	-
Marriage of daughter	49	49	-	-	-
Change of trade/line of work	49	-	-	8.84	-
Dowry-related problems in family	47	51	-	-	-
Shifting house many times in same station	46	39	-	5.14	-
Wife starting a job	42	25	26	-	-
Pay fine/minor violation of law	40	48	-	6.05	2.66
Difficulty with seniors	39	52	-	12.21	2.59
Conflict with friends in unit	38	52	-	12.21	2.57
Promotion/outstanding achievement	38	37	-	5.39	-
Wife leaving the job	37	25	26	-	-
Lack of son	37	51	-	-	-
Failing in promotion cadre/ examination	36	43	-	13.52	2.84
Going on posting	23	33	-	8.52	-

* Life change units scored on a scale of 0–20

** Life change units scored on a scale of 1–5

### Norms of the AFMC LES

Demographic variables of the subjects included in the study are given in Table 4. The mean number of life events (rounded off to the nearest whole number) experienced by service personnel in the past year and in their life-time is 4 and 13, respectively. Norms of the number of life events in past one year and life-time are given in Tables 5 and 6, respectively. Norms of the LCU scores for past one year and life-time are given in Tables 7 and 8, respectively. Comparison of life events with age, length of service, rank, marital status and domicile are given in Table 9.

**Table 4:** Demographic characteristics of the subjects

Variable (*n*=600)	Army-Field (*n*=600)	Army-Peace (*n*=100)	Air Force (*n*=100)	Navy
Age (in years)				
Mean	33.59	33.14	33.62	31.21
SD	8.24	8.23	6.95	4.81
Service (in years)				
Mean	14.08	13.87	14.91	11.62
SD	7.85	7.30	7.72	5.86
Marital status				
Married	528	516	87	71
Unmarried	72	84	13	29
Origin				
Rural	376	399	36	41
Urban	224	221	64	59
Rank				
Sepoy	265	258	48	49
Naik	114	126	19	19
Havaldar	165	152	21	20
Junior Commissioned Officer/Officer	56	64	12	12

**Table 5:** Life events in past one year

Sample	*n*	Mean	SD	25th percentile	Median	75th percentile
Army-Field	600	4.18	2.90	2	4	6
Army-Peace	600	3.54	3.34	2	3	5
Air Force	100	3.43	3.03	1	3	5
Navy	100	3.58	1.90	2	3	5
Armed Forces	1400	3.86	3.17	2	3	5

KW test: p>0.5 No significant differences between the four groups of subjects on number of life events in past one year

**Table 6:** Life events in life-time

Sample	*n*	Mean	SD	25th percentile	Median	75th percentile
Army-Field	600	13.18	7.41	9	12	16
Army-Peace	600	11.81	6.92	7	11	15
Air Force	100	12.49	5.20	10	11	16
Navy	100	11.66	3.96	9	12	14
Armed Forces	1400	12.56	6.84	8	12	16

KW test: p>0.05 No significant differences between the four groups of subjects on number of life events in life-time

**Table 7:** Life change unit (LCU) scores in past one year

Sample	*n*	Mean	(SD)	25th percentile	Median	75th percentile
Army-Field	600	119.11	(99.17)	46	102	171
Army-Peace	600	118.26	(99.23)	46	100	165
Air Force	100	89.90	(102.70)	40	64	78
Navy	100	99.37	(61.16)	62	100	153
Armed Forces	1400	114.68	(96.28)	46	100	171

KW test: No significant differences between scores of Army personnel in field and peace. Scores of Army personnel in peace and field significantly more than Air Force and Navy. No significant differences between Air Force personnel and Navy personnel. No significant difference between Army personnel in field and peace.

**Table 8:** Life change unit (LCU) scores in life-time

Sample	*n*	Mean	(SD)	25th percentile	Median	75th percentile
Army-Field	600	650.03	(387.52)	356	551	892
Army-Peace	600	513.67	(243.90)	332	493	668
Air Force	100	619.11	(235.39)	467	683	799
Navy	100	480.02	(178.35)	217	434	470
Armed Forces	1400	576.46	(297.57)	341	503	781

KW test: Scores of Army personnel in field significantly more than Army personnel in peace and Navy. Scores of Army personnel in field not significantly different from Air Force, but significantly more than Army personnel in peace and Navy personnel. Scores of Air Force personnel significantly more than Army personnel in peace and Navy personnel. Scores of Army personnel in peace significantly more than Navy personnel.

**Table 9:** Comparison of life events and demographic variables

Variable	*n*	Life events in past year		Life events in life-time	
			
		Mean	(SD)	Mean	(SD)
Age					
	≤35 years	4.00	(3.02)	11.03	(5.73)
	>35 years	3.86	(3.43)	14.70	(5.15)*
Length of service					
	≤10 years	4.23	(3.21)	9.84	(5.32)
	>10 years	3.62	(3.16)	14.06	(5.13)*
Rank					
	Sepoy/Lance Naik	3.71	(2.32)	11.16	(6.12)
	Naik	4.16	(3.87)	13.68	(5.91)
	Havaldar	3.82	(3.16)	13.87	(6.00)
	Junior commissioned officer/officer	4.05	(3.00)	13.16	(4.07)
Marital status					
	Married	3.85	(3.27)	13.26	(5.65)*
	Unmarried	3.95	(2.76)	8.33	(4.10)
Domicile					
	Rural	3.99	(3.50)	11.91	(5.63)
	Urban	3.66	(2.99)	11.51	(5.09)

* Mann–Whitney test: p<0.05, significant

*Comparison of weightage given to each life event in respect of Army, Navy and Air Force personnel:* Analysis did not reveal any significant differences in the weightage assigned to different life events by personnel of the three services.

### Reliability

*Cronbach's alpha:* Internal consistency for the AFMC LES is 0.861, which is quite high.^18^ In the item total statistics (Table 10) examining the last column, it is seen that the alpha would drop if any of the items were to be deleted from the scale. That is, all the items contribute to making the internal consistency of the scale high.

**Table 10:** Cornbach's alpha SPSS output (Item-Total Statistics)

Item	Corrected item-total correlation	Cornbach's alpha if item deleted
1	0.435	0.855
2	0.357	0.856
3	0.165	0.860
4	0.195	0.859
5	0.339	0.857
6	0.202	0.859
7	0.357	0.856
8	0.403	0.855
9	0.355	0.856
10	0.430	0.855
11	0.244	0.858
12	0.217	0.859
13	0.336	0.857
14	0.278	0.858
15	0.316	0.857
16	0.378	0.856
17	0.271	0.858
18	0.291	0.858
19	0.461	0.854
20	0.263	0.858
21	0.287	0.858
22	0.168	0.859
23	0.304	0.857
24	0.345	0.857
25	0.221	0.859
26	0.194	0.859
27	0.293	0.857
28	0.309	0.857
29	0.275	0.858
30	0.226	0.859
31	0.310	0.857
32	0.222	0.859
33	0.300	0.857
34	0.435	0.854
35	0.240	0.858
36	0.137	0.861
37	0.313	0.857
38	0.349	0.856
39	0.177	0.859
40	0.337	0.857
41	0.278	0.858
42	0.383	0.856
43	0.353	0.857
44	0.394	0.856
45	0.451	0.854
46	0.352	0.856
47	0.276	0.858
48	0.258	0.858
49	0.373	0.856
50	0.211	0.859
51	0.250	0.858
52	0.326	0.857

*Split-half correlation and reliability:* Correlation between Half 1 and half 2 were 0.748 and are significant (p < 0.01). Equal length Spearman-Brown coefficient was 0.856. Guttman split-half coefficient was 0.855. These are within acceptable limits.^18^

*Test–retest reliability:* Test–retest reliability of the scale has been calculated by administration of the scale a second time to the same group of subjects after a gap of 7 days. There was a significant positive correlation between the past year scores (rho=0.945, *n*=200, p < 0.01, two-tailed) and life-time scores (rho=0.968, *n*=200, p < 0.01, two-tailed).

### Validity

*Criterion validity:* There is at present no other scale to measure stressful life events in Armed Forces personnel. Hence, the PSLES was used as a predictor of stressful life events. The AFMC LES demonstrated significant correlations with many of the personality factors. On the PSLES, administered to the subjects in the same sitting along with the AFMC LES, the mean number of life events in one year and life-time were 2.82 (S.D. 2.01) and 10.72 (S.D. 3.64), respectively. The AFMC life events scale and the PSLES had a significant positive correlation in life-time scores (Spearman's rho=0.726, *n*=200, p < 0.01, two-tailed) and also past one year scores (Spearman's rho=0.49, *n*=200, p < 0.01, two-tailed).^18^

*Content validity:* No measuring instrument in the behavioral sciences would be of any application unless its contents permitted a fairly representative sample of behavioural characteristics. From this viewpoint the items for the AFMC LES were taken initially from consensus of experts in the field (psychiatrists and clinical psychologists). Face validity of items was taken into consideration and items were drawn from the common experience of service personnel. The favorable opinion of experts argues well for content validity. In addition, in the second version of the scale 3 open-ended questions were included to enable respondents to report any other stressful event, which was not included in the scale. Thus, the respondents were able to suggest new items or modifications to specific items, which were taken into consideration while preparing the final version of the scale.

### Factor analysis

The total variance explained by principle component analysis is shown in Table 11. Thirteen distinct clusters (factors) of life events were extracted by factor analysis. These clusters may be characterized according to the nature of the items contained within them. Although not all the items within each cluster relate specifically to these headings, the major contributing items in terms of strengths of their intercorrelations are identified by this nomenclature. The loadings on the subsets of events under each factor are given in Table 12.

**Table 11:** Total variance explained

	Initial eigenvalues			Extraction sums of squared loadings			Rotation sums of squared		
			
Component	Total	% of variance	Cumulative %	Total	% of variance	Cumulative %	Total	% of variance	Cumulative %
1	7.826	15.049	15.049	7.826	15.049	15.049	3.691	7.099	7.099
2	3.727	7.167	22.216	3.727	7.167	22.216	3.297	6.340	13.439
3	2.411	4.636	26.852	2.411	4.636	26.852	2.573	4.948	18.387
4	1.845	3.549	30.400	1.845	3.549	30.400	2.371	4.560	22.947
5	1.737	3.341	33.742	1.737	3.341	33.742	2.191	4.213	27.160
6	1.399	2.690	36.431	1.399	2.690	36.431	2.134	4.103	31.263
7	1.358	2.611	39.042	1.358	2.611	39.042	1.736	3.338	34.601
8	1.288	2.478	41.520	1.288	2.478	41.520	1.715	3.297	37.898
9	1.224	2.354	43.874	1.224	2.354	43.874	1.602	3.081	40.979
10	1.144	2.200	46.075	1.144	2.200	46.075	1.563	3.006	43.985
11	1.078	2.073	48.147	1.078	2.073	48.147	1.550	2.981	46.966
12	1.021	1.963	50.110	1.021	1.963	50.110	1.494	2.872	49.839
13	1.000	1.924	52.034	1.000	1.924	52.034	1.142	2.196	52.034
									
14	0.976	1.876	53.911						
15	0.959	1.844	55.754						
16	0.933	1.794	57.548						
17	0.924	1.776	59.325						
18	0.905	1.741	61.066						
19	0.865	1.663	62.728						
20	0.844	1.623	64.352						
21	0.805	1.549	65.900						
22	0.790	1.519	67.419						
23	0.787	1.513	68.932						
24	0.768	1.478	70.410						
25	0.754	1.451	71.861						
26	0.732	1.407	73.268						
27	0.723	1.391	74.658						
28	0.711	1.368	76.026						
29	0.694	1.335	77.361						
30	0.686	1.319	78.680						
31	0.660	1.269	79.949						
32	0.646	1.243	81.192						
33	0.638	1.227	82.420						
34	0.629	1.210	83.629						
35	0.613	1.179	84.808						
36	0.598	1.150	85.958						
37	0.580	1.115	87.073						
38	0.573	1.102	88.175						
39	0.564	1.085	89.260						
40	0.536	1.031	90.292						
41	0.517	0.995	91.286						
42	0.502	0.965	92.251						
43	0.498	0.957	93.208						
44	0.477	0.918	94.127						
45	0.460	0.885	95.012						
46	0.445	0.855	95.867						
47	0.437	0.841	96.708						
48	0.410	0.788	97.497						
49	0.373	0.717	98.214						
50	0.347	0.666	98.880						
51	0.307	0.589	99.470						
52	0.276	0.530	100.000						

Extraction Method: Principal ComponentAnalysis

**Table 12:** Results of a principal component factor analysis with varimax rotation of AFMC LES

Item No.	Items	Loading	% of variance
*Factor 1. Severe domestic and job crises*			
26.	Divorce from wife	0.772	15.049
27.	Conflict with family members	0.569	
28.	Not receiving salary because of debit	0.528	
29.	Arranging for a big loan	0.557	
30.	Court martial	0.820	
31.	Black ink entry	0.621	
32.	Pay fine	0.574	
33.	Hospitalization due to serious illness	0.591	
*Factor 2. Operational stress*			
8.	Completing a tenure in operational area	0.688	7.167
9.	Participation in divisional exercises	0.636	
10.	Completing a tenure of field posting	0.721	
38.	Spending tenure of high altitude posting	0.673	
46.	Fighting enemies during war	0.405	
47.	Fighting terrorists	0.677	
*Factor 3. Separation from significant others*			
5.	Child getting a job	0.533	4.636
11.	Child leaving town for higher education	0.420	
19.	Receiving medal for bravery	0.471	
44.	Marriage of daughter	0.724	
48.	Going abroad on duty	0.688	
49.	Release from service	0.404	
*Factor 4. Change in employment status of family members*			
14.	Spouse having illicit relations	0.442	3.549
15.	Wife starting a job	0.631	
16.	Wife leaving the job	0.691	
22.	Winning a lottery	0.540	
*Factor 5. Family formation and promotion at work*			
1.	Getting married	0.767	3.341
2.	Birth of child	0.798	
7.	Passing promotion cadre	0.514	
45.	Constructing own house	0.357	
*Factor 6. Inter-personal problem*			
17.	Sex-related problems	0.443	2.690
23.	Sanctioned leave being cancelled	0.479	
34.	Death of a close relative	0.290	
41.	Difficulty with seniors	0.714	
42.	Conflict with friends in unit	0.513	
43.	Dowry-related problems in family	0.434	
*Factor 7. Denial*			
3.	Shifting house many times in same station	0.461	2.611
6.	Child not getting admission in school	0.493	
25.	Annual leave not being sanctioned	0.551	
36.	Not getting government accommodation	0.408	
*Factor 8. Job and physical crises*			
20.	Failing in promotion cadre	0.514	2.478
37.	Going on posting within 48 hours	0.580	
50.	Amputation of body parts	0.553	
*Factor 9. Punishments*			
4.	Loss of identity card	0.390	2.354
35.	Red ink entry	0.798	
40.	Demotion	0.412	
*Factor 10. Sanctioning of leave*			
24.	Sanction of casual leave	0.751	2.200
52.	Sanction of annual leave	0.812	
*Factor 11. Change in work role*			
18.	Receiving medal in sports	0.530	2.073
39.	Change of trade	0.659	
51.	Receiving highest marks in firing	0.388	
*Factor 12. Procreation issues*			
12.	Wife not conceiving for long duration	0.708	1.963
13.	Lack of son	0.764	
*Factor 13. Posting*			
21.	Going on posting	0.513	1.924

## DISCUSSION

An extensive literature, varying from anecdotal reports, intensive psychoanalytic studies, controlled comparisons, to large-scale surveys, attest to the importance that is generally assigned to life events in the genesis of many diseases. In fact, researchers have established a positive relationship between stressful life events and a number of physical and psychiatric illnesses.^15^,^19^–^21^

Life events questionnaires have in recent times been widely used as potential indices of presumptive stress. It is assumed that people seldom forget major life events and usually do not deny them. Memories of external events are not as prone to distortion as are assessments of subjective mood, emotion or capacity to function. A quantitative measure of presumptive stress can be derived. Each event can be assigned a weight and the weights can be summed to measure the quantity of stress that an individual has undergone. As indicated in the results, 29 of the 52 AFMC LES items (55.8%) were not included in the SRRS, while 24 of the 52 AFMC LES items (46.2%) were not included in the PSLES. An inspection of these items shows that they help to capture unique aspects of military life that are particularly likely to produce stress in service personnel. This is a major strength of the AFMC LES. During the construction of the AFMC LES open-ended questions were also utilized to obtain suggestions about new items from respondents. The use of open-ended questions has been recommended by several authorities^16^,^22^ and made the construction of our scale methodologically sound.

### Measurement of stress over different time periods

In view of the findings that recall of events in recent time period is better than relatively remote events and also the problem of retrospective contamination, it was decided to follow the method adopted by Singh *et al.*^16^ and keep 2 time scales: (i) life-time, and (ii) past one year. Time scale of one year was chosen and not 6 months as it is indicated in many studies^22^ that with 6 months as cut-off period it would be difficult in many psychiatric disorders to differentiate between the event being a symptom or result of the disorder in contrast to the event being a cause of the disorder, e.g. loss of job can be a cause of depression and sometimes depression can result in loss of job. The total number of stressful life events being experienced in past year and life-time in the present study are higher when compared to PSLES. Service personnel undergo more number of life events in past one year (*n*=4) and in total life-span (*n*=13) as compared to civilian counterparts. The probable explanation may be that a number of items such as sanction of casual leave, divisional-level exercises being common occurrences could have influenced the score. Concurrent validity is evident from the fact that the mean scores on present scale were highly correlated with PSLES scores.

### Consistency of scale

An important issue for this scaling is the amount of variability. If this scale is to be suitable for wider application there must be at least a moderate consensus between individuals as to the perceived stressfulness of events. One way of evaluating the degree of consensus is to consider the variability of event judgement in the total sample. In the AFMC LES the standard deviations of LCU of life events ranged from extremes of 32.73 to 21.16; most were between 25 and 31. When compared with the scale range these standard deviations appear moderate in magnitude. They certainly indicate good confidence limits for the population means.

### Quantification of stressful life events

Measurement of the magnitude of life events is a major methodological problem in life events research. An apparently simple solution to this problem was offered in the SSRS.^22^ A simple form of this procedure, magnitude estimation, involves designating a modulus with an assigned value and asking judges to rate other stimuli in relation to this modulus. Holmes and Rahe designated ‘marriage’ as the modulus, assigned it a value of 500, and obtained quantitative judgements about the amount of change or readjustment in relation to it for each of the other 42 events on their list. LCU scores based on these ratings have been presented as a measure of the stressfulness of the rated events.^23^ If we weigh events in terms of their different LCU scores and pay attention to how these weights add up when a series of events occurs, the risk of illness attached to the events will vary directly with the magnitude of LCU scores. Holmes in particular has emphasized the high level of consensus about the amount of change associated with each life event. He refers to correlations in the 0.80s and 0.90s between the mean ratings for each event obtained from such diverse status groups as blacks and Japanese as well as whites. This is contrary to our findings.

Holmes' argument for the universalism of perception of the stressfulness of particular life events, however, has been sharply criticized. Two of Holmes' collaborators have pointed out that considerable group differences are masked by the reported correlations. Ratings secured in Sweden were consistently higher than Holmes' American ratings. Certain differences in ratings of events by Japanese and American judges seemed to be related to differences between the two cultures. Other researchers have also reported cultural contrasts.^22^ Sharp differences were found in the way rural sample and urban sample ranked such events as ‘marriage’.^23^ In the urban sample, for example, marriage is ranked 4th in contrast with 21st in the rural sample in terms of the amount of change involved. These differences are meaningful in terms of contrasts in the norms and customs of the two samples. Zheng and Lin^24^ from China also reported that the rank order of stressfulness of several stressful life events differed significantly across cultures. The findings of the above studies are in agreement with our findings.

The highest weightage of 83 LCUs in the present study was assigned to ‘wife having illicit relations with other person’. This finding differs from western studies but concurs with the finding of PSLES as Indian civilians in that study also gave a high weightage of 82. Next highest LCU score of 80 was assigned to court martial followed by amputation of body parts and divorce from wife (Table 1). Court martial is probably the most undesirable event in the life of service personnel and apart from loss of face among the peer group may involve punishment and even also loss of job. The assessment of the service personnel on items of divorce from wife, sexual problems, marriage of a daughter is very similar to the PSLES (Table 2). Lack of son was given a mean stress score of 51 in PSLES while our soldiers have given it a lesser mean score of 37. Probably more awareness of gender equality in respect of children is imbibed by them through education in the services.

Another interesting observation is that trouble with seniors is again having a lesser mean score as compared to civilian counterparts. The soldiers mean score on this item is 39 whereas the PSLES mean score is 52 (Table 2). This is probably because in the Armed Forces conflict is generally resolved within the members of the unit by the superiors. The item death of a close relative in the present study was given a mean stress score of 58. In the PSLES, the item has a higher mean score of 66. Sudden unexpected deaths of comrades are not infrequent in a serviceman's life. This might have, to a certain extent, blunted the perception of change in relation to this item. Child leaving home for higher education involves environmental or financial changes. This has more stress score as compared to the western culture where the weightage assigned is only 29 on SRRS (Table 2). Service personnel and Indian civilians have assigned a weighted score of 49 to marriage of a daughter and arranging for a big loan. On the contrary, the western population assigns a lower weightage of 30. Probably financing is easier with the help of funding agencies in the West. Apart from this aspect, the individuals' earnings are also more in the West.^17^

Among the items unique to service personnel, fighting against enemy during war has a weightage of 69. Greater stresses and strains mark combat situations. During combat death of self and comrades is a distinct possibility. In addition, there is physical exhaustion. The reasons for high mean scores are of course self-explanatory. Loss of identity card is also assigned a high mean score of 69. Loss of identity card is viewed as negligence and loss of government property, which is a serious offence, and invariably attracts disciplinary action. Fighting against terrorists has a mean score of 60. Servicemen have clubbed this with routine items such as constructing own house and birth of child. This is a reflection of the difficult times we are passing through.^17^ It is worth mentioning here that sanctioning of casual leave has a mean score of 43 while sanction of annual leave has a mean score of 34. The difference can be explained by the fact that annual leave is generally planned whereas casual leave is on the basis of requirement hence LCUs being perceived are more. Other routine items such as going on posting are not perceived as having much of change.

### Categorization of life events

Scale items were further categorized into desirable items (*n*=25) and undesirable items (*n*=27). There was no significant difference in the stress experienced on desirable items (mean=57.89; SD=7.55) compared to undesirable items (mean=53.42; SD=14.99). This observation is not in agreement with the findings of Singh *et* al.^16^ The probable reason for this is that while the PSLES contained 10 desirable and 32 undesirable items, the present scale is balanced and contains almost equal number of desirable and undesirable events, which is again an improvement on the PSLES.

### Demographic characteristics of the subjects

It is apparent from Table 4 that the samples drawn from Army, Air Force and Navy differ in certain respects. The age and service of Navy sample is less than that of the others. Of the Navy sample, 29% are bachelors compared to about 15% of the rest. Army sample appears to be predominantly rural (36%–37%) while the Air Force and Navy sample is predominantly urban (56%–57.5%). However, the distribution according to rank was similar in all three samples. The aims of the present study were the construction of a stressful life events scale and standardize it on a sample of service personnel. Therefore, these differences in demographic characteristics do not vitiate the results of the present study.

### Number of life events experienced in past year and life-time

Norms as obtained in the present study for male armed forces personnel of life events for one year are 3.86±3.17 and for life-time it is 12.56±6.84. These norms are much higher than the norms of PSLES of 1.90± 2.62 for one year and 10.34±5.40 for life-time. It is obvious that the present scale reflects the increased number of life events experienced by service personnel. In addition, there were no significant differences in the number of life events experienced in past year and life-time by personnel from Army, Navy and Air Force. It indicates that the scale can be applied to personnel from all the three services.

### Norms for LCU scores in past year

There was no significant difference in LCU scores between Army personnel in field (LCU=119.11) and peace (118.26). The LCU scores of Army personnel were significantly more than those for Air Force and Navy personnel probably due to the fact that at present the major burden of operational deployment is on the Army personnel. No significant differences were observed in LCU scores in past year of Air Force personnel and Navy personnel (Table 7).

### Norms for LCU scores in life-time

Army personnel in field have significantly higher LCU scores in life-time compared to Army personnel in peace and Navy personnel but not Air Force personnel (Table 8). The reason for this is not very clear. It may be related to the way the samples were collected or it may reflect the fact that life in the Navy is less stressful. It must be mentioned here that the small sample size of Air Force and Navy personnel in the present study precludes a firm conclusion that has to await a larger study.

There was no significant difference in number of life events in past year experienced by service personnel 35 years of age and less compared to those more than 35 years of age (Table 9). This is probably due to the fact that all service personnel are facing similar environment. However, those more than 35 years of age experienced statistically significantly more life-time life events as compared to those aged 35 years and less (Table 9). This probably is a reflection of accumulation of life events with age and is in agreement with the findings of Zheng and Lin.^24^ Individuals with more than 10 years of service had significantly more life events in their life-time compared to those with service of 10 years or less though there was no statistically significant difference between the two groups in the life events in the past year (Table 9). This finding is probably a reflection of accumulation of life events with age. There was no statistically significant difference in the scores of life events in past year experienced and in life-time by all ranks (Table 9). This indicates that all personnel are facing the same stressors.

Married individuals experienced less life events in past one year than unmarried subjects, but the difference was not statistically significant (Table 9). On the other hand, married subjects experienced significantly more life events in life-time as compared to the unmarried (Table 9). It is seen that marriage and the life events related to spouse and children occur only in married personnel. Hence the fact that the number of life events in life-time is significantly more in married personnel is expected. Life events experienced in past year and life-time by subjects from a rural background was somewhat more than those from the urban background (Table 9). However, the difference was not statistically significant. This again is a reflection of the fact that all personnel are sharing the same environment with similar stressors.

The 13 factors identified in the present study may be used as a stepping-stone for the development of multiple scale inventories of life events. By increasing the number of events quite reliable scales could be developed for each specific content domain. Also, by careful selection of events under each category, one may increase the representativeness and generalizability of the scales. The sampling of events should consider such factors as the life stage of the individual being assessed, fateful versus personal failure events, desirable versus undesirable events, objective versus subjective events. It is likely that separate norms should be used for individuals stratified by age, marital status, and social class. Thus, by carefully increasing the number of events in each homogeneous category multiple scale inventories could be developed.

## LIMITATIONS

The sample of the present study included only male personnel. With ladies joining the services in increasing numbers, future studies should include female armed forces personnel also. The Army, Air Force and Naval personnel were not matched for age. Further, only normal personnel were studied and patients with various stress-related physical and psychiatric disorders were not studied. The future direction of the study is to include Paramilitary Force and Police Force personnel of both sexes to evaluate the applicability of the scale to all security force personnel.

## CONCLUSION

The AFMC LES includes the unique experiences of service personnel that are not included in any life events scale available in India or the west. It was observed that certain life events are common to service personnel and civilian counterparts. The scale has adequate reliability and validity. There is internal consistency in the scale with the routine items being rated very low. On the AFMC LES, normal service personnel face about 4 life events in past one year and about 13 life events in life-time. Normal service personnel face about 114.68 LCUs in past one year and about 576.46 LCUs of stress in life-time.

## Appendices

**APPENDIX A:** A FMC LIFE EVENTS SCALE

Name:	Rank:	Age:	Sex:	Education:	Date:	
Domicile: Rural/Urban	Total service:	Trade:	Marital status:	No. of children:	boys/ girls

**Directions**

A list of common events which occur in our life is given below. Please read the events carefully and tick those items which have occurred in the past year in the ‘Past one year’ column. Some items may have occurred sometime in your life-time. Please tick against those items in the ‘Life-time’ column.

**Table:**

Item No.	Life event	Past one year	Life-time
1.	Getting married		
2.	Birth of child		
3.	Shifting house many times in same station		
4.	Loss of identity card		
5.	Child getting a job		
6.	Child not getting admission in school		
7.	Passing the promotion cadre		
8.	Completing a tenure in operational area		
9.	Participation in divisional exercises		
10.	Completing a tenure of field posting		
11.	Child leaving town for higher education		
12.	Wife not conceiving for long duration		
13.	Lack of son		
14.	Wife/husband having illicit relations		
15.	Wife starting a job		
16.	Wife leaving the job		
17.	Sex-related problems		
18.	Receiving medal in sports		
19.	Receiving medal for bravery during war		
20.	Failing in promotion cadre		
21.	Going on posting		
22.	Winning a lottery		
23.	Sanctioned leave being cancelled		
24.	Sanction of casual leave		
25.	Annual leave not being sanctioned		
26.	Divorce from wife		
27.	Conflict with family members		
28.	Not receiving salary because of debit		
29.	Arranging for a big loan		
30.	Court martial		
31.	Black ink entry		
32.	Pay fine		
33.	Hospitalization due to serious illness		
34.	Death of a close relative		
35.	Red ink entry		
36.	Not getting government accommodation		
37.	Going on posting within 48 hours		
38.	Spending tenure of high altitude posting		
39.	Change of trade		
40.	Demotion		
41.	Difficulty with seniors		
42.	Conflict with friends in unit		
43.	Dowry-related problems in family		
44.	Marriage of daughter		
45.	Constructing own house		
46.	Fighting against enemies during war		
47.	Fighting against terrorists		
48.	Going abroad on duty		
49.	Getting release from service		
50.	Amputation of body parts		
51.	Receiving highest marks in firing		
52.	Sanction of annual leave		

## References

[1] Dohrenwend BS, Dohrenwend BP (1978). Some issues in research on stressful life events. J Nerv Ment Dis.

[2] Swank LR (1949). Combat exhaustion. J Nerv Ment Dis.

[3] Silver RC, Holman EA, McIntosh DN (2002). Nationwide longitudinal study of psychological responses to September 11. JAMA.

[4] Rafanelli C, Roncuzzi R, Milaneschi Y (2005). Stressful life events, depression and demoralization as risk factors for acute coronary heart disease. Psychother Psychosom.

[5] Kozora E, Ellison MC, Waxmonsky JA (2005). Major life stress, coping styles, and social support in relation to psychological distress in patients with systemic lupus erythematosus. Lupus.

[6] Horan WP, Ventura J, Nuechterlein KH (2005). Stressful life events in recent-onset schizophrenia: Reduced frequencies and altered subjective appraisals. Schizophr Res.

[7] Sandberg S, Jarvenpaa S, Penttinen A (2004). Asthma exacerbations in children immediately following stressful life events: A Cox's hierarchical regression. Thorax.

[8] Schanberg LE, Gil KM, Anthony KK (2005). Pain, stiffness, and fatigue in juvenile polyarticular arthritis: Contemporaneous stressful events and mood as predictors. Arthritis Rheum.

[9] Dhanaraj M, Rangaraj R, Arulmozhi T (2005). Nonepileptic attack disorder among married women. Neurol India.

[10] Bay E, Hagerty B, Williams RA (2005). Chronic stress, salivary cortisol response, interpersonal relatedness, and depression among community-dwelling survivors of traumatic brain injury. J Neurosci Nurs.

[11] Kendler KS, Kuhn JW, Vittum J (2005). The interaction of stressful life events and a serotonin transporter polymorphism in the prediction of episodes of major depression: A replication. Arch Gen Psychiatry.

[12] Portzky G, Audenaert K, van Heeringen K (2005). Suicide among adolescents. A psychological autopsy study of psychiatric, psychosocial and personality-related risk factors. Soc Psychiatry Psychiatr Epidemiol.

[13] Holmes TH, Rahe RH (1967). The Social Readjustment Rating Scale. J Psychosom Res.

[14] Paykel ES, Prusoff BA, Uhlenhuth EH (1971). Scaling of life events. Arch Gen Psychiatry.

[15] Pestonjee DM (1997). Stress and coping. The Indian experience.

[16] Singh G, Kaur D, Kaur H (1984). Presumptive Stressful Life Events Scale—A new stressful life events scale for use in India. Indian J Psychiatry.

[17] Raju MSVK, Srivastava K, Chaudhury S (2001). Quantification of stressful life events in service personnel. Indian J Psychiatry.

[18] Kline TJB (2005). Psychological testing. A practical approach to design and testing.

[19] Rubertsson C, Wickberg B, Gustavsson P (2005). Depressive symptoms in early pregnancy, two months and one year postpartum—prevalence and psychosocial risk factors in a national Swedish sample. Arch Women Mental Health.

[20] Wurtman RJ (2005). Genes, stress, and depression. Metabolism.

[21] Basu J, Basu S, Bhattacharyya S (2004). Ego functions in relation to stressful life events and indices of psychopathology in paranoid schizophrenia. Psychol Rep.

[22] Dohrenwend BP (1998). Adversity, stress, and psychopathology.

[23] Miller FT, Bentz WK, Aponte JR, Dohrenwend BS, Dohrenwend BP (1974). Stressful life events: Their nature and effects.

[24] Zheng Y-P, Lin K-M (1994). A nationwide study of stressful life events in mainland China. Psychosom Med.

